# ATG7-enhanced impaired autophagy exacerbates acute pancreatitis by promoting regulated necrosis via the miR-30b-5p/CAMKII pathway

**DOI:** 10.1038/s41419-022-04657-4

**Published:** 2022-03-07

**Authors:** Liang Ji, Zhi-hong Wang, Yu-hua Zhang, Yi Zhou, De-sheng Tang, Chang-sheng Yan, Jia-min Ma, Kun Fang, Lei Gao, Nian-sheng Ren, Long Cheng, Xiao-yu Guo, Bei Sun, Gang Wang

**Affiliations:** 1 Key Laboratory of Hepatosplenic Surgery, Ministry of Education, No. 23 Youzheng Street, Nangang District, 150001 Harbin, Heilongjiang China; 2 Department of Breast Surgery, The First Affiliated Hospital of Harbin Medical University, No. 23 Youzheng Street, Nangang District, 150001 Harbin, Heilongjiang China; 3 Department of Thyroid Surgery, The First Hospital of China Medical University, No. 155 Nanjing North Street, Heping District, 110001 Shenyang, Liaoning China; 4 Department of Hepatopancreatobiliary Surgery, Cancer Hospital of the University of Chinese Academy of Sciences (Zhejiang Cancer Hospital), No. 1 Banshan Road, Gongshu District, 310022 Hangzhou, Zhejiang China; 5 Institute of Basic Medicine and Cancer (IBMC), Chinese Academy of Sciences, No. 1 Banshan Road, Gongshu District, 310022 Hangzhou, Zhejiang China; 6 Department of Pancreatic and Biliary Surgery, The First Affiliated Hospital of Harbin Medical University, No. 23 Youzheng Street, Nangang District, 150001 Harbin, Heilongjiang China

**Keywords:** Cytokines, Acute inflammation

## Abstract

The present study was performed to explore whether and how impaired autophagy could modulate calcium/calmodulin-dependent protein kinase II (CAMKII)-regulated necrosis in the pathogenesis of acute pancreatitis (AP). Wistar rats and AR42J cells were used for AP modeling. When indicated, genetic regulation of CAMKII or ATG7 was performed prior to AP induction. AP-related necrotic injury was positively regulated by the incubation level of CAMKII. ATG7 positively modulated the level of CAMKII and necrosis following AP induction, indicating that there might be a connection between impaired autophagy and CAMKII-regulated necrosis in the pathogenesis of AP. microRNA (miR)-30b-5p was predicted and then verified as the upstream regulator of CAMKII mRNA in our setting of AP. Given that the level of miR-30b-5p was negatively correlated with the incubation levels of ATG7 after AP induction, a rescue experiment was performed and indicated that the miR-30b-5p mimic compromised ATG7 overexpression-induced upregulation of CAMKII-regulated necrosis after AP induction. In conclusion, our results indicate that ATG7-enhanced impaired autophagy exacerbates AP by promoting regulated necrosis via the miR-30b-5p/CAMKII pathway.

## Introduction

Because the exact underlying mechanism is still far from known and no specific therapy has yet been developed, acute pancreatitis (AP) remains a clinical challenge with considerable morbidity and mortality [1]. Several cell death pathways are evoked and play sophisticated roles in the pathogenesis of AP. Unlike apoptosis, which acts as a self-defense mechanism against AP-related injuries, necrosis usually correlates positively with the severity of AP. For two decades, necrosis was thought to be an accidental passive cell death in response to physiochemical insults. However, increasing evidence has suggested that there might be a series of signaling pathways involved in the regulation of emerging programmed cell death named regulated necrosis [2–4]. Regulated necrosis is defined as a genetically controlled cell death process that eventually results in cellular leakage, and it is morphologically characterized by cytoplasmic granulation, as well as organelle and/or cellular swelling [3]. Regulated necrosis usually consists of necroptosis, pyroptosis, parthanatos, ferroptosis and others. Among these regulated programs, only necroptosis has been relatively well elucidated by the canonical receptor-interacting protein kinase 1 (RIPK1)/RIPK3/mixed lineage kinase domain-like protein pathway. We must admit that there remain many unknowns in the understanding of regulated necrosis. Previously, our results suggested that the inhibition of RIPK1-dependent regulated necrosis provided protection against AP via the RIPK1/NF-κB/aquaporin (AQP) 8 pathway, indicating a new horizon for exploring the mechanism of AP and its possible targeted therapy [5].

Autophagy is a quality control process that serves as a salvage mechanism for recycling cytoplasmic materials and preserving energy via lysosome-driven degradation in response to a series of extracellular and intracellular stresses, including nutrient deprivation, hormonal therapy, pathogenic infection, misfolded proteins and damaged organelles [6]. Fundamental or physiological autophagy is an essential cellular self-aid behavior in a harsh environment and otherwise will lead to cell damage. Two early-phase characteristics in pancreatic acini during AP have long been noticed, namely, the accumulation of cytoplasmic vacuoles and the premature activation of trypsinogen. Recently, evidence has accumulated that both of these pathological responses to AP could be attributed to impaired autophagy, although the potential mechanism is still a debate [7–11]. Our previous report suggested that impaired autophagy in our AP rat model resulted from overactivation of the upstream formation of autophagosomes via the AMPK/mTOR pathway in response to increased levels of incubated hydrogen sulfide [7]. The molecular machinery of autophagy is referred to as ATG genes. ATG7 acts as an ubiquitin-activating enzyme, is required for ATG12–ATG5 conjugation and the subsequent formation of autophagosomes [12].

Intracellular Ca^2+^ homeostasis is critical for many vital biological processes during AP. In our previous report, the level of intracellular Ca^2+^ was positively correlated with the extent of cellular energy stress and necrosis after AP induction [13]. Calcium/calmodulin-dependent protein kinase II (CAMKII) is a multifunctional serine/threonine protein kinase that mediates the phosphorylation of its substrates in response to cytoplasmic Ca^2+^ increase. Therefore, CAMKII could be considered as an indicator of intracellular Ca^2+^ levels in the setting of AP [14, 15]. Furthermore, CAMKII was found to be indispensable during trypsinogen activation in the AP model induced by nicardipine [15]. In addition to the phosphorylation of its substrate, intersubunit, intraholoenzyme autophosphorylation of CAMKII develops after the entry of Ca^2+^ into acinar cells and acquires autonomous and Ca^2+^-independent activity [16]. Therefore, CAMKII might be a promising target in the future management of AP. However, there remain some unrevealed issues before concluding the abovementioned clinical significance of CAMKII. Both the upstream mechanism of CAMKII alteration and the downstream effects imposed on cell death pathways in the setting of AP are unknown according to the only related report [15].

MicroRNAs (miRs) are a group of evolutionarily highly conserved single‐stranded nucleotides of 19–25 nt that negatively regulate gene expression in a sequence-specific manner at the post-transcriptional level [17]. Several miRs are believed to be involved in the pathogenesis of AP through the underlying mechanisms consisting of their regulation of the cell death pathway, local inflammatory response and targeted organ injury [18]. miR-21-5p could regulate necroptosis, a type of regulated necrosis, through a protein inhibitor of the activated STAT 3/STAT3 pathway in AP [19]. The miR-30 family, consisting of miR-30a, miR-30b, miR-30c, miR-30d, miR-30e, and miR-384, is believed to be sophisticated in many inflammatory conditions [20, 21].

The present study was performed to explore whether and how impaired autophagy could modulate the expression of CAMKII and regulated necrosis in the pathogenesis of AP, and thus to shed new light on the future interpretation and management of AP.

## Materials and methods

### Reagents

Sodium taurocholate (Na-TC) and sodium pentobarbital were purchased from Sigma-Aldrich (St. Louis, MO, USA). Lentiviral vectors encoding ATG7 (Lv-ATG7) or CAMKII (Lv-CAMKII) and lentivirus with scrambled sh-ATG7 (Lv-sh-ATG7) or sh-CAMKII (Lv-sh-CAMKII) were purchased from GeneChem (Shanghai, China). MiR-30b-5p mimic and mimic negative control were purchased from RiboBio (Guangzhou, China). The primary antibodies used for Western blot were microtubule-associated protein 1 light chain 3 (LC3), p62, ATG7 and high mobility group protein B1 (HMGB1) purchased from Cell Signaling Technology (Danvers, MA, USA), tumor necrosis factor α (TNF-α), β-actin and interleukin-1β (IL-1β) purchased from Santa Cruz Biotechnology (Dallas, Texas, USA), CAMKII purchased from Abcam (Shanghai, China), and lysosome-associated membrane protein-2 (LAMP-2) purchased from Thermo Fisher Scientific (Rockford, IL, USA).

### Model establishment and ethics statement

Sixty male Wistar rats, weighing 200–250 g, were supplied by the Animal Research Center at the First Affiliated Hospital of Harbin Medical University (Harbin, China). The rat model of AP was established using a previously described method [7, 13]. Briefly, the rats were anaesthetized by an intraperitoneal injection of sodium pentobarbital (40 mg/kg). Then, a midline laparotomy was performed, and the distal pancreaticobiliary duct was ligated. AP was induced by a retrograde infusion of 3.5% Na-TC (0.15 ml/100 g) into the pancreaticobiliary duct. The rats in sham group were subjected to laparotomy alone. The animal care and experimental protocols were all approved by the Institutional Animal Care and Use Committee of Harbin Medical University and conducted in accordance with the Guide for the Care and Use of Laboratory Animals.

### Experimental design in vivo

The rats were fed rodent chow and water ad libitum in an environmentally controlled room (18–21 °C, 40–60% relative humidity, 12 h light/dark cycle). After 1 week of acclimatization, the rats were deprived of food overnight before the experiments. Based on our preliminary experiments, an injection of knockdown (10^6^ TU/mL in 200 μL of PBS) or overexpression (5 × 10^6^ TU/mL in 200 μL of PBS) lentivirus vector through the caudal vein 5 days before the experiment was performed to regulate the level of ATG7 or CAMKII in rats. In sham and AP groups, an equivalent volume of the corresponding negative control was administered instead. All of the surviving rats in each group were sacrificed at 6 h after AP induction. For every single blood sample, serum was obtained after centrifugation at 3000 r/min for 15 min and then stored at −80 °C until assayed. Each pancreatic sample was homogeneously prepared for three different purposes: rinsed in saline buffer and snap-frozen in liquid nitrogen at −80 °C for Western blot, fixed in 4% buffered paraformaldehyde for 48 h and then embedded in paraffin for hematoxylin and eosin staining (H&E staining) and immunohistochemistry (IHC), fixed in 2 mL of 2.5% glutaraldehyde and postfixed in 1% osmium tetroxide solution for transmission electron microscopy (TEM).

### Measurement of parameters in serum and pancreas

The serum levels of C-reactive protein (CRP) and amylase were spectrophotometrically measured using a biochemical autoanalyzer (Toshiba, Tokyo, Japan) as previously described [5, 13]. The serum levels of TNF-α and IL-1β were measured using enzyme-linked immunosorbent assay kits (R&D Systems, Minneapolis, MN, USA) according to the manufacturer’s instructions. The pancreatic levels of malondialdehyde (MDA), myeloperoxidase (MPO) and lipid peroxidase (LPO) were measured using specific kits (Jiancheng, Nanjing, China) according to the manufacturer’s instructions.

### H&E staining

H&E staining was performed to observe the level of inflammation and tissue damage under a light microscope. Two professional pathologists who were blinded to the experimental protocol scored the pancreatic tissue on a scale from 0 to 4 for the degrees of edema, inflammation, hemorrhage and necrosis in 20 randomly selected fields. We applied the scoring system defined by Kusshe et al. [22] and the final score for each group was totaled.

### Transmission electron microscopy (TEM)

The fixed samples were dehydrated through a graded series of ethanol and embedded in epoxy resin. Ultrathin sections (80 nm) were collected on copper grids, double-stained with uranyl acetate and lead citrate, and then examined under a Hitachi H-7100 transmission electron microscope (Hitachinaka, Japan) at 80 kV. For quantification, the percentage of autophagic vacuoles (mono- or bilayer membrane structures wrapped around the partially degraded cargos) per cytoplasmic area was calculated on each print [6].

### IHC

The protocol for IHC has been previously described [5, 7]. In short, the specimens were dewaxed and incubated with 3% H_2_O_2_ in methanol at 37 °C for 10 min to quench endogenous peroxidase. After blocking at room temperature for 30 min, the sections were incubated with CAMKII (1: 200) overnight at 4 °C. Subsequently, the sections were incubated with secondary antibodies (1:200; ZSGB-BIO, Beijing, China) and developed for color with diaminobenzidine peroxidase color development kits (ZSGB-BIO). Finally, the sections were counterstained with hematoxylin. The sections were observed under a light microscope and the expression of protein was quantified by integrated optical density (IOD) with Image-Pro Plus v6.0 software (Media Cybernetics, Crofton, MA, USA) in 20 randomly selected fields. The cells with the presence of a dark reddish-brown chromogen indicate a positive signal.

### Cell cultures

The rat pancreatic exocrine cell line AR42J was purchased from the American Type Culture Collection (Manassas, VA, USA) and cultured in Dulbecco’s modified Eagle’s medium (Gibco, Grand Island, NY, USA) supplemented with 10% fetal bovine serum (ScienCell, San Diego, CA, USA), 100 U/mL penicillin and 100 mg/ml streptomycin (Invitrogen, Carlsbad, CA, USA) at 37 °C in a 5% CO_2_ humidified incubator.

### Experimental design in vitro

AR42J cells were seeded into 6-well plates (5 × 10^4^ per well) until 70% confluence so that appropriate volumes of lentivirus could be added to achieve the multiplicity of the infection value recommended by the manufacturer. After lentiviral infection, stable clones were selected with 2 μg/mL puromycin (Sigma-Aldrich) for 2–4 weeks. Alternatively, transfection of the miR-30b-5p mimic (100 nM) was conducted to manipulate the genetic level of miR-30b-5p. The efficiency of all transfections was evaluated by quantitative real-time polymerase chain reaction (qRT-PCR) and/or Western blot. To stimulate AP in vitro, AR42J cells were incubated with 500 μM Na-TC for 3 h as detailed in our previous reports [7, 13]. For control group, the cells were treated with an equivalent volume of PBS to that of Na-TC administered for AP induction.

### Adenosine triphosphate (ATP) assays

The ATP contents of pancreatic tissues or cells were measured using an Enhanced ATP Assay Kit (Beyotime, Beijing, China) according to the manufacturer’s instructions, normalized to the protein concentration and finally expressed as nmol/mg.

### Mitochondrial transmembrane potential (MTP) assay

Intracellular MTP was determined using the dual-emission mitochondrial dye 5,5′,6,6′-tetrachloro-1,1′,3,3′-tetraethylbenzimidazolocarbocyanine iodide (JC-1, Beyotime) as detailed elsewhere [13]. In short, staining was performed using 2.5 μg/mL JC-1 for 15 min at 37 °C. After staining, the cells were rinsed three times with PBS. Dye equilibration was allowed for 10 min at room temperature prior to imaging. Fluorescent images of the emissions at 529 and 590 nm were captured using a laser confocal microscope (Carl Zeiss, Oberkochen, Germany). JC-1 exhibits a fluorescence emission shift upon aggregation from 529 nm (green monomer, indicative of low MTP) to 590 nm (red J-aggregates, indicative of high MTP). Thus, a reduced ratio of red/green fluorescence indicates mitochondrial depolarization.

### Measurement of intracellular Ca^2+^ concentration

The method for the measurement of intracellular Ca^2+^ concentration has been described previously [13]. In brief, the cells were preloaded with 5 μM Fura-2 AM (Beyotime) in HEPES buffer for 1 h at room temperature. Images of the Fura-2-loaded cells were captured using a laser confocal microscope (Carl Zeiss) and analyzed using Image-Pro Plus v6.0 software. Background-subtracted fluorescent images for excitation at 340 and 380 nm were captured. The intracellular Ca^2+^ concentration was estimated from the ratio of Fura-2 fluorescence emitted at 510 nm after excitation at 340 nm to that after excitation at 380 nm, according to the Grynkiewicz equation [23].

### Necrosis assay

Cell necrosis was detected by an apoptosis and necrosis assay kit (Beyotime) according to the manufacturer’s instructions. Briefly, 10^5^ cells were seeded in 6-well plate and then treated according to the study design. The cells were subjected to the mixed solution consisting of 2 mL dying buffer, 10 μL Hoechst 33342 solution and 10 μL propidium iodide (PI) solution for 30 min at 4 °C. The cells were washed with PBS twice before imaging. The images were acquired using a confocal laser scanning microscope (Carl Zeiss), and the average percentages of necrotic cells (suggested by a duo-fluorescence of red and blue) were calculated in five randomly selected high-power fields.

### RNA isolation, reverse transcription, and qRT-PCR

Total RNA extraction and reverse transcription were performed as previously described [5]. qRT-PCR (SYBR Green Assay, Roche, Mannheim, Germany) was performed on Applied Biosystem 7500. Data analysis was performed using the 2^−△△CT^ method. U6 was used as the internal reference for miR-30b-5p, and GADPH was used as the internal reference for CAMKII mRNA. The primer sequences were designed by Primer 5.0 and are listed in Table 1.

**Table 1:** Primer sequence of genes.

Gene	Primer sequence
CAMKII	F: 5′-GACAAGAAAACTCCGCAA-3′
	R: 5′-AAATCAACCCCAAAATCC-3′
GADPH	F: 5′-TGGAGTCTACTGGCGTCTT-3′
	R: 5′-TGTCATATTTCTCGTGGTTCA-3′
miR-30b-5p	F: 5′-ACACTCCAGCTGGGTGTAAACATCCTACAC-3′
	R: 5′-CTCAACTGGTGTCGTGGAGTCGGCAATTCAGTTGAGAGCTGAGT-3′
U6	F: 5′-CTCGCTTCGGCAGCACA-3′
	R: 5′-AACGCTTCACGAATTTGCGT-3′

Primer sequence of CAMKII, GADPH, miR-30b-5p and U6.

*F* forward primer sequence, *R* reverse primer sequence.

### Western blot

The Western blot protocol has been previously described [5, 7, 13]. In brief, pancreatic tissues or cells were homogenized in protein lysate buffer that contained protease inhibitor and phosphatase inhibitor (Roche, Shanghai, China), and debris was removed by centrifugation. The samples were resolved on polyacrylamide sodium dodecyl sulfate gels and electrophoretically transferred to polyvinylidene difluoride membranes. The membranes were blocked with 5% skimmed milk and incubated with the proper primary antibodies (1:1000) and horseradish peroxidase-conjugated secondary antibodies (1:2000, ZSGB-BIO). Immunostained bands were detected using enhanced chemiluminescence kits (Pierce Chemical, Rockford, IL, USA). β-actin (1:1000) was used as the protein loading control.

### Autophagy flux assay

To facilitate the autophagy flux assay, AR42J cells were transfected with a mRFP-GFP-LC3 tandem lentivirus (GeneChem) according to the manufacturer’s instructions [7]. Theoretically, GFP is a stably folded protein and relatively resistant to lysosomal proteases. However, the low pH level inside the lysosomes quenches the fluorescent signal of GFP. With this construct, autophagosomes and autolysosomes were labeled with yellow (mRFP and GFP) and red (mRFP only). After treatments, the images of differently allocated cells were captured using a confocal laser scanning microscope (Carl Zeiss) and analyzed using Image-Pro Plus v6.0 software. We calculated the ratio of autolysosomes (red) to autophagosomes (yellow) per cell to evaluate the status of autophagy flux.

### Luciferase reporter assay

To determine whether CAMKII acts as a direct target of miR-30b-5p, the 3′-untranslated region (3′-UTR) of wild-type CAMKII (WT) and mutant-CAMKII (MUT) were amplified and then cloned into the pmiR-RB-Report^TM^ vector (RiboBio). For the luciferase reporter assay, AR42J cells were cotransfected with 50 nM miR-30b-5p or 50 nM miR-control. Luciferase activity was determined using the dual luciferase assay system (Promega, Madison, WI, USA) after 48 h of transfection. Luciferase activity was normalized to Renilla luciferase activity.

### Statistical analysis

Data are presented as the mean±standard deviation of at least three independent experiments and were analyzed using SAS 9.1 for Windows (SAS Institute, Cary, NC, USA). The data were analyzed using one-way ANOVA followed by a Scheffe test. A *P* value of <0.05 was considered statistically significant.

## Results

### AP-related necrotic injury was positively regulated by the incubation level of CAMKII

**Fig. 1:**
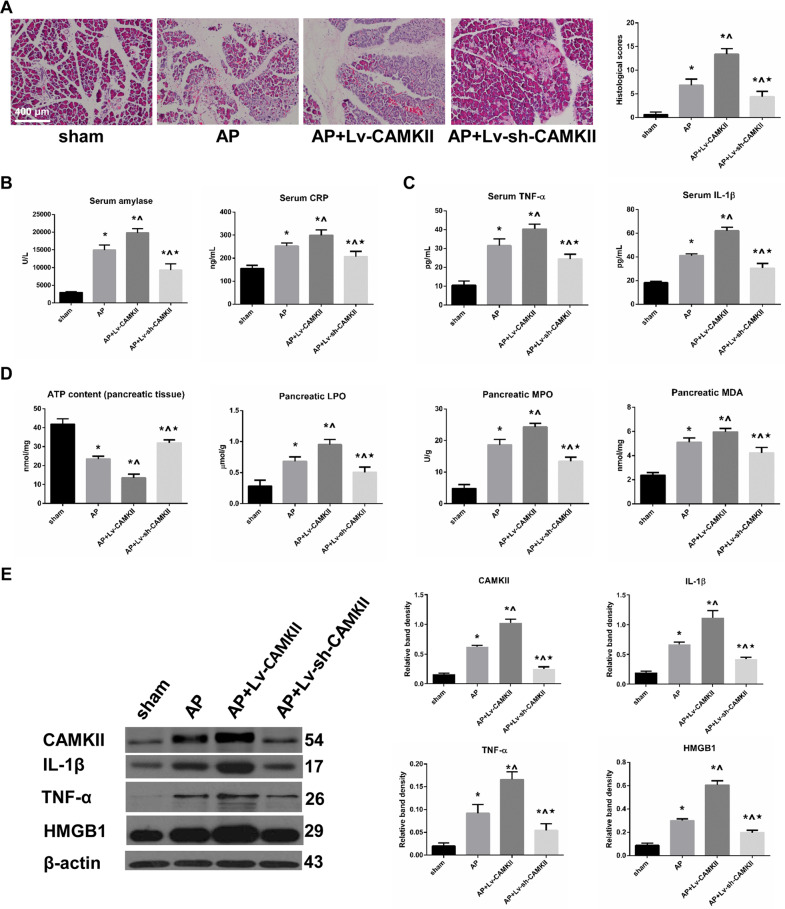
AP-related necrotic injury was positively regulated by the incubation level of CAMKII. **A** Representative photographs and histological scores of HE-stained pancreatic sections harvested from the rats that were subjected to sham operation, AP, AP + Lv-CAMKII and AP + Lv-sh-CAMKII for 6 h since AP induction. Bar = 400 μm. **B**, **C** Serum levels of amylase, CRP, TNF-α, and IL-1β in rats as described above. **D** Pancreatic levels of ATP contents, LPO, MPO, and MDA in rats as described above. **E** Representative Western blot images and quantifications of CAMKII, HMGB1, TNF-α and IL-1β protein expression in pancreatic tissues harvested from the rats as described above. β-actin was used as the protein loading control. Data were presented as mean ± SD (*N* ≥ 3). ^**∗**^*P* < 0.05 versus sham, ^**^**^*P* < 0.05 versus AP, and *P < 0.05 versus AP + Lv^−^CAMKII. AP acute pancreatitis, ATP adenosine triphosphate, CAMKII calcium/calmodulin-dependent protein kinase II, CRP C-reactive protein, HE hematoxylin-eosin, HMGB1 high mobility group protein B1, IL-1β interleukin-1β, LPO lipid peroxidase, MDA malonic dialdehyde, MPO myeloperoxidase, SD standard deviation, TNF-α tumor necrosis factor-α.

The pathomorphological alterations of pancreatic tissues that were subjected to sham, AP, AP + Lv-CAMKII or AP + Lv-sh-CAMKII were observed by H&E staining. A series of necrosis, hemorrhage, edema and inflammatory cell infiltration developed after AP induction, whereas no obvious abnormality was found in sham group. Necrosis was more severe in AP + Lv-CAMKII group and less severe in AP + Lv-sh-CAMKII group than that in AP group. The histological scores were significantly increased after AP induction in comparison to those in sham group. In addition, CAMKII overexpression before AP induction was associated with an increase in histological scores, whereas CAMKII knockdown before AP was associated with a decrease in histological scores compared to that in AP group (Fig. 1A). The measurements of the contents of inflammatory cytokines and other AP-related parameters were in accordance with the findings of H&E staining (Fig. 1B–E and Supplementary Fig. 1). Therefore, AP-related necrotic injury was positively regulated by the incubation level of CAMKII.

### The extent of AP-related overactivated autophagy was positively regulated by the incubation level of ATG7

**Fig. 2:**
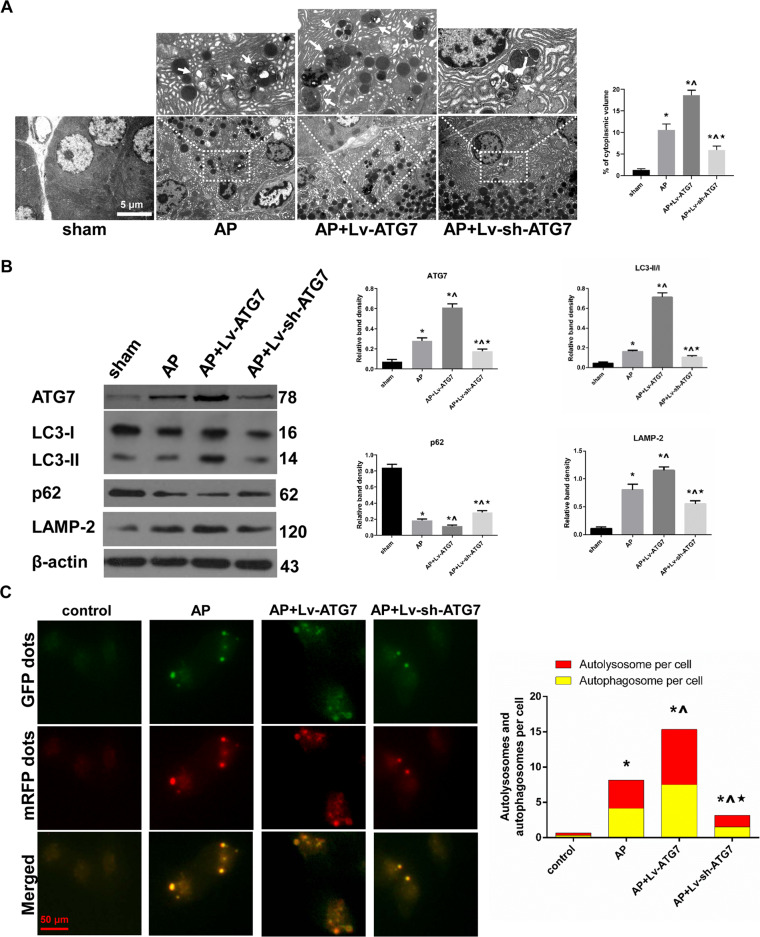
The extent of AP-related overactivated autophagy was positively regulated by the incubation level of ATG7. **A** Representative TEM photos of pancreatic tissues harvested from the rats that were subjected to sham operation, AP, AP + Lv-ATG7 and AP + Lv-sh-ATG7 for 6 h since AP induction. The percentage of autophagic vacuoles (white arrows) per cytoplasm area was calculated. Bar = 5 μm. **B** Representative Western blot images and quantifications of ATG7, p62, LAMP-2 protein expression and LC3 conversion in pancreatic tissues as described above. β-actin was used as the protein loading control. **C** Representative fluorescent photographs of autophagy flux assay in mRFP-GFP-LC3 tagged AR42J cells that were subjected to control, AP, AP + Lv-ATG7 and Lv-sh-ATG7 for 3 h since AP induction. The number of autophagic dots and ratio of red dots (autolysosomes) to yellow dots (autophagosomes) per cell was calculated. Bar = 50 μm. Data were presented as mean ± SD (*N* ≥ 3). ^**∗**^*P* < 0.05 versus sham or control, ^**^**^*P* < 0.05 versus AP, and *P < 0.05 versus AP + Lv^−^ATG7. AP acute pancreatitis, CAMKII calcium/calmodulin-dependent protein kinase II, LAMP-2 lysosome-associated membrane protein-2, LC3 microtubule-associated protein 1 light chain 3, SD standard deviation, TEM transmission electron microscopy.

Previously, we reported that autophagy was overactivated in our AP model of rats induced by Na-TC [7]. In the present study, we performed TEM to examine the cytoplasmic accumulation of autophagic vacuoles. The percentage of autophagic vacuoles per cytoplasmic area was significantly increased after AP induction, compared with that in sham group. Moreover, ATG7 overexpression significantly potentiated the percentage of autophagic vacuoles per cytoplasmic area, whereas ATG7 knockdown significantly decreased the percentage of autophagic vacuoles per cytoplasmic area compared with that in AP group (Fig. 2A). Moreover, AP induction was associated with an increase in LC3 conversion (LC3 II/I) and a decrease in p62. These alterations could be potentiated by prior ATG7 overexpression to AP induction or compromised by prior ATG7 knockdown to AP induction (Fig. 2B and Supplementary Fig. 2). To rule out the possibility of disordered autophagosome-lysosome fusion or lysosome-driven degradation, a Western blot to determine the expression of LAMP-2 (an indispensable lysosome membrane protein for autophagosome–lysosome fusion) in vivo and an autophagy flux assay to determine the ratio of autolysosomes to autophagosomes per cell in vitro were introduced into the present study. Our results indicated that neither the maturation of autophagosomes nor lysosome-driven degradation was disordered after AP induction, with or without ATG7 modulation (Fig. 2B, C and Supplementary Fig. 2). Additionally, the counts of autophagic dots (autophagosomes and autolysosomes) per cell in the autophagy flux assay in vitro were in accordance with the cytoplasmic accumulation of autophagic vacuoles in TEM observations in vivo. Therefore, our results suggested that the extent of AP-related overactivated autophagy was positively regulated by the incubation level of ATG7.

### ATG7 positively modulated the levels of CAMKII and necrosis following AP induction

**Fig. 3:**
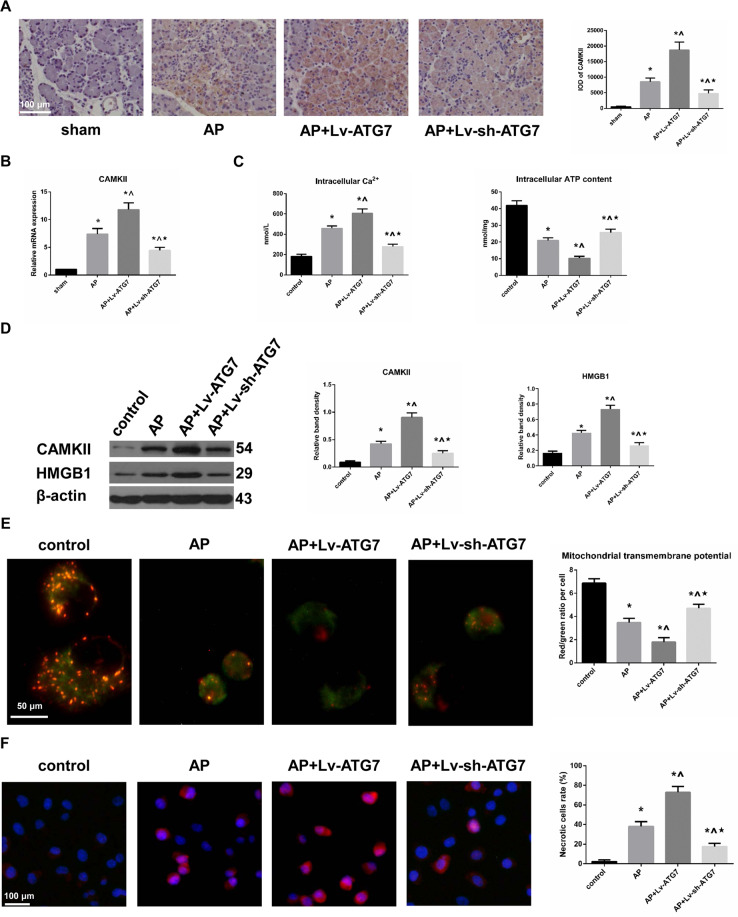
ATG7 positively modulated the levels of CAMKII and necrosis following AP induction. **A** Representative IHC photographs of CAMKII expression in pancreatic tissues harvested from the rats as described in Fig. 2A. The corresponding IODs were analyzed with Image-Pro Plus v6.0 software. Bar = 100 μm. **B** Levels of CAMKII mRNA in pancreatic tissues harvested from the rats as described in Fig. 2A. **C** Intracellular contents of ATP and Ca^2+^ in cells that were subjected to control, AP, AP + Lv-ATG7 and Lv-sh-ATG7 for 3 h since AP induction. **D** Representative Western blot images and quantifications of CAMKII and HMGB1 protein expression in cells mentioned above. β-actin was used as the protein loading control. **E** Representative fluorescent images indicative of MTP by JC-1 staining in cells mentioned above, and the ratio of red/green fluorescence was calculated to indicate MTP. Bar = 50 μm. **F** Representative fluorescent images indicative of necrosis and apoptosis by PI-Hoechst 33342 duo-staining in cells mentioned above, and the necrotic cells (indicated by a duo-fluorescence of red and blue) rate was calculated. Bar = 100 μm. Data were presented as mean ± SD (*N* ≥ 3). ^**∗**^*P* < 0.05 versus sham or control, ^**^**^*P* < 0.05 versus AP, and *P < 0.05 versus AP + Lv^−^ATG7. AP acute pancreatitis, ATP adenosine triphosphate, CAMKII calcium/calmodulin-dependent protein kinase II, HMGB1 high mobility group protein B1, IHC immunohistochemistry, IOD integrated optical density, MTP mitochondrial transmembrane potential, PI propidium iodide, SD standard deviation.

The pancreatic mRNA and protein expression of CAMKII were evaluated by QT-PCR and IHC. Our results indicated that both the mRNA and protein expression of CAMKII were increased in AP group compared to sham group. Moreover, prior ATG7 overexpression to AP induction upregulated both the mRNA and protein expression of CAMKII, whereas prior ATG7 knockdown to AP induction downregulated both the mRNA and protein expression of CAMKII compared to that in AP group (Fig. 3A, B). In vitro, the protein expression of CAMKII detected by Western blot when AR42J cells were subjected to control, AP, AP + Lv-ATG7 and AP + Lv-sh-ATG7 treatments echoed the in vivo results (Fig. 3D and Supplementary Fig. 3). The intracellular concentration of Ca^2+^, which acts as the upstream stimulus of CAMKII, was measured in vitro. AP induction was associated with an increased intracellular concentration of Ca^2+^ in comparison to that in control group. Prior ATG7 overexpression to AP induction potentiated the intracellular concentration of Ca^2+^ but prior ATG7 knockdown to AP induction decreased the intracellular concentration of Ca^2+^, compared to that in AP group (Fig. 3C). By doing so, we can conclude that ATG7 positively modulated the level of CAMKII. MTP assay using JC-1 and measurements of intracellular ATP contents indicated that there was a functional deficiency in mitochondria after AP induction, and its extent was positively correlated with the incubated level of CAMKII (Fig. 3C, E). To directly determine the effect of CAMKII in regulating necrosis when cells were subjected to control, AP, AP + Lv-ATG7 and AP + Lv-sh-ATG7 treatments, necrosis assays and protein expression of HMGB1 (a well-known member of damage-associated molecular patterns released from the nuclei of necrotic cells) using Western blotting were performed. Our results indicated that necrosis was positively associated with the incubation level of ATG7 (Fig. 3D, F and Supplementary Fig. 3). Therefore, these findings suggested that ATG7 positively modulated the levels of CAMKII and necrosis following AP induction.

### ATG7 overexpression promoted the activation of CAMKII via miR-30b-5p inhibition

**Fig. 4:**
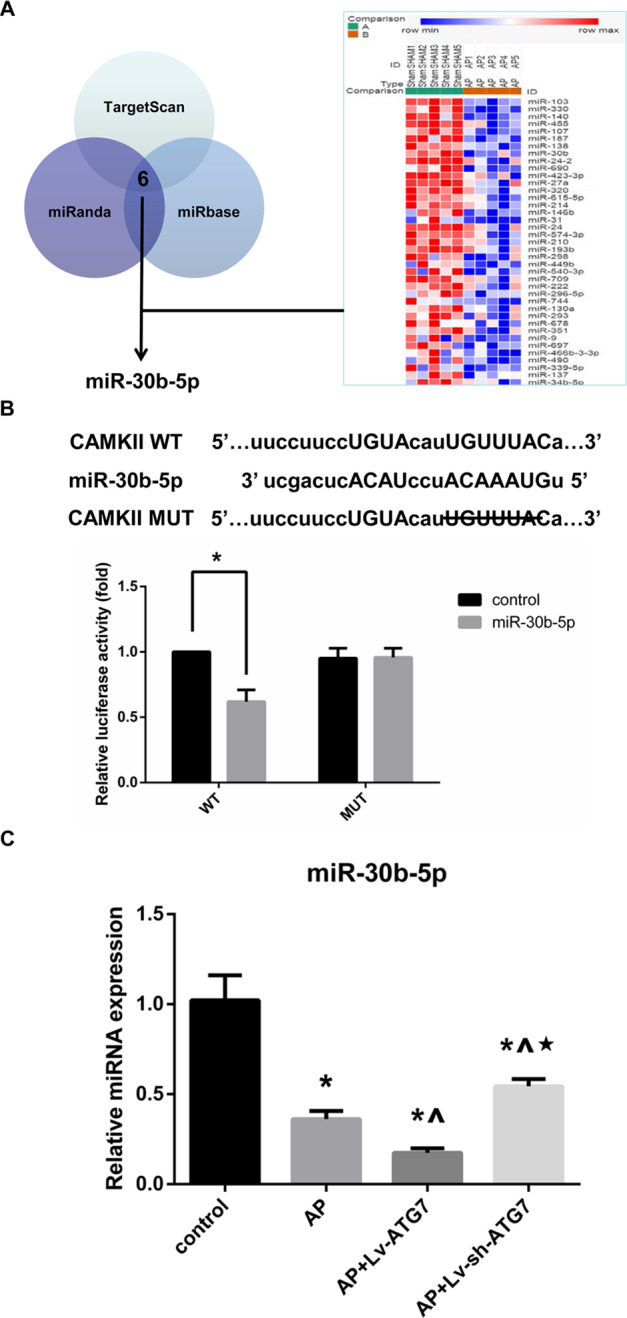
ATG7 overexpression promoted the activation of CAMKII via miR-30b-5p inhibition. **A** An online bioinformatic analysis and a microarray analysis of miRNA using five pairs of pancreatic tissues of rats with or without AP modeling were performed to predict the potential upstream miR targeting CAMKII mRNA. **B** Relative luciferase activities of the reporters harboring the wild-type (WT) or the mutated (MUT) CAMKII 3′-untranslated region (UTR) that were subjected to miR-30b-5p mimic or negative control. **C** Relative miR-30b-5p expressions in cells that were subjected to control, AP, AP + Lv-ATG7 and Lv-sh-ATG7 for 3 h since AP induction. Data were presented as mean ± SD (*N* ≥ 3). ^**∗**^*P* < 0.05 versus control, ^**^**^*P* < 0.05 versus AP, and *P < 0.05 versus AP + Lv-ATG7. AP acute pancreatitis, CAMKII calcium/calmodulin-dependent protein kinase II, SD standard deviation.

To determine the underlying upstream miR targeting CAMKII in our settings of AP, an online bioinformatic analysis using TargetScan, miRanda and miRbase was first performed. There were six overlapping miRs that bind to the 3′-UTR of CAMKII mRNA in theory: miR-30-5p/384-5p, miR-122-5p, miR-135-5p, miR-145-5p, miR-203-3p and miR-551b-5p. A microarray analysis of miRNA using five pairs of pancreatic tissues of rats with or without AP modeling was performed. Thirty-nine miRs were significantly decreased (fold change < −2, *P* < 0.05) due to AP modeling. A second overlap between the results of online prediction and microarray analysis was conducted and indicated that miR-30b-5p (a family member of miR-30-5p/384-5p) might serve as the upstream miR in regulating the expression of CAMKII (Fig. 4A). To confirm this hypothesis, we cloned a wild-type or a mutated CAMKII 3′-UTR downstream of the luciferase reporter gene and then performed a luciferase reporter assay. We found that exogenous miR-30b-5p expression significantly decreased the activity of the reporter harboring the wild-type CAMKII 3′-UTR, whereas there was no significant change in the activity of the reporter harboring the mutated CAMKII 3′-UTR (Fig. 4B). Subsequently, we noticed that the level of miR-30b-5p was significantly decreased when AR42J cells were subjected to AP induction. In addition, the level of miR-30b-5p was significantly increased when ATG7 knockdown was performed before AP induction and significantly decreased when ATG7 overexpression was performed before AP induction, compared with that in AP group (Fig. 4C). Therefore, our results indicated that ATG7 overexpression promoted the activation of CAMKII via miR-30b-5p inhibition.

### The miR-30b-5p mimic compromised ATG7 overexpression-induced upregulation of CAMKII-regulated necrosis

**Fig. 5:**
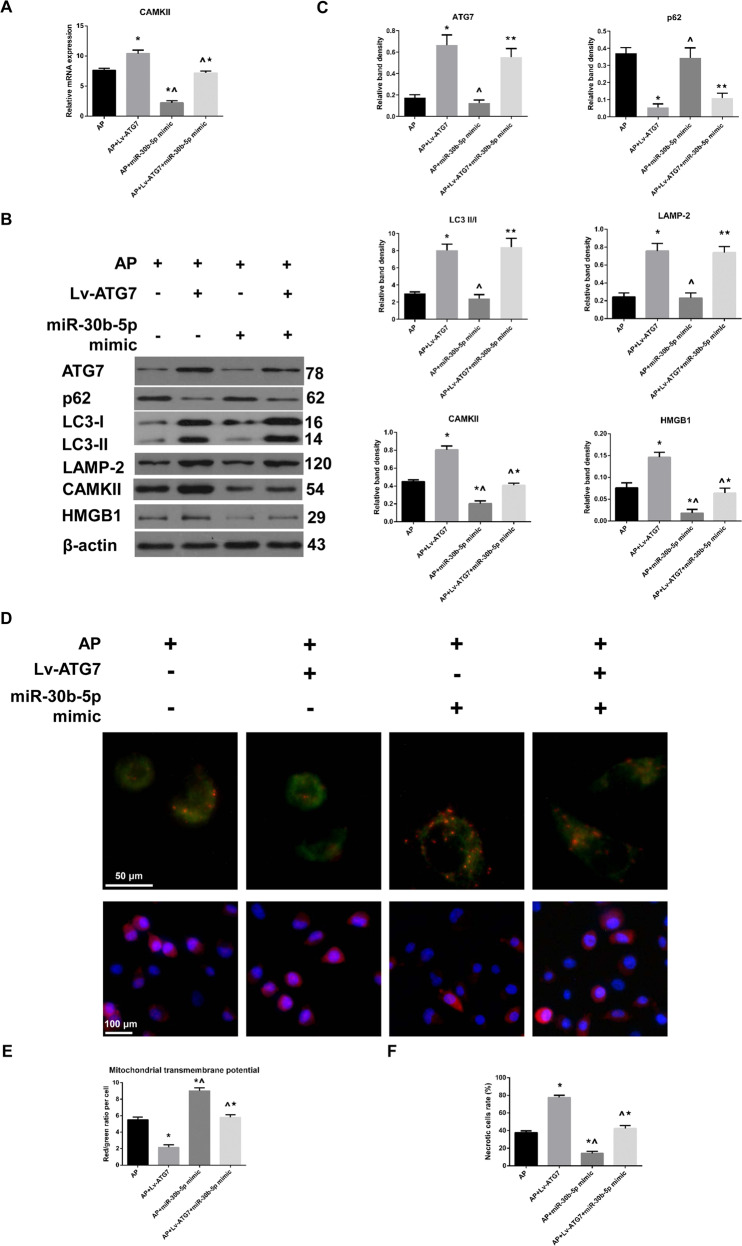
The miR-30b-5p mimic compromised ATG7 overexpression-induced upregulation of CAMKII-regulated necrosis. **A** Levels of CAMKII mRNA in cells that were subjected to AP, AP + Lv-ATG7, AP + miR-30b-5p mimic and AP + Lv-ATG7 + miR-30b-5p mimic. **B**, **C** Representative Western blot images (**B**) and quantifications (**C**) of ATG7, p62, LAMP-2, CAMKII, HMGB1 protein expression and LC3 conversion in cells as described above. β-actin was used as the protein loading control. **D**–**F** Representative fluorescent images indicative of MTP by JC-1 staining (**D**, upper panel, Bar = 50 μm) and those indicative of necrosis and apoptosis by PI-Hoechst 33342 duo-staining (**D**, lower panel, Bar = 100 μm) in cells mentioned above. The ratio of red/green fluorescence was calculated to indicate MTP (**E**). The necrotic cells (indicated by a duo-fluorescence of red and blue) rate was calculated (**F**). Data were presented as mean ± SD (*N* ≥ 3). ^**∗**^*P* < 0.05 versus AP, ^**^**^*P* < 0.05 versus AP + Lv-ATG7, and *P < 0.05 versus AP + miR-30b-5p mimic. AP acute pancreatitis, CAMKII calcium/calmodulin-dependent protein kinase II, HMGB1 high mobility group protein B1, LAMP-2 lysosome-associated membrane protein-2, LC3 microtubule-associated protein 1 light chain 3, MTP mitochondrial transmembrane potential, PI propidium iodide, SD standard deviation.

To confirm whether the impaired autophagy induced by ATG7 overexpression could upregulate CAMKII-regulated necrosis via miR-30b-5p inhibition. AR42J cells were subjected to AP, AP + Lv-ATG7, AP + miR-30b-5p mimic and AP + Lv-ATG7 + miR-30b-5p mimic. In accordance with previous findings in the present study, prior ATG7 overexpression to AP induction was not only associated with increased mRNA and protein expression of CAMKII, but also associated with enhanced autophagy, as indicated by decreased expression of p62 and increased LC3 conversion and expression of LAMP-2, compared to AP induction alone. Pre-treatment with the miR-30b-5p mimic in addition to AP induction was associated with a decrease in CAMKII in comparison to that in AP group. More importantly, the expression of CAMKII in AP + Lv-ATG7 + miR-30b-5p mimic group was significantly increased compared to that in AP + miR-30b-5p mimic group but significantly decreased compared to that in AP + Lv-ATG7 group (Fig. 5A–C and Supplementary Fig. 4). The levels of HMGB1 by Western blot, MTP and necrosis assay were performed to check the alterations of CAMKII-regulated necrosis. Our results indicated that the MTP loss and necrosis rate in AP + Lv-ATG7 + miR-30b-5p mimic group were significantly increased compared to those in AP + miR-30b-5p mimic group but significantly decreased compared to those in AP + Lv-ATG7 group (Fig. 5D, E). The same result could be seen when it referred to alterations in the level of HMGB1 (Fig. 5B, C and Supplementary Fig. 4). Therefore, we can conclude that the miR-30b-5p mimic compromised ATG7 overexpression-induced upregulation of CAMKII-regulated necrosis.

## Discussion

AP continues to be a clinical challenge for which no specific treatment has been developed thus far due to its sophisticated pathogenesis. When pancreatic acinar cells are doomed following the attack of AP, several major cell death pathways can be activated, namely, apoptosis, necrosis and autophagy. In contrast to apoptosis acting as a protective cell death pathway, necrosis always acts as a detrimental process and correlates well with the severity of AP. Necrosis is considered as a rapid passive process with no specific signaling control for a long period. However, regulated necrosis, an emerging concept, challenges our past understanding of necrosis. Cell death research was thus revitalized by the understanding that necrosis could occur in a highly regulated and genetically controlled manner [2, 3, 24]. Given that the extent of pancreatic acinar cell necrosis is positively associated with the severity and poor prognosis of AP, the role of regulated necrosis and its potential mechanisms in the pathogenesis of AP are worthy of elucidation. In our previous study, our findings suggested that inhibition of RIPK1-dependent regulated acinar cell necrosis provides protection against AP via the RIPK1/NF-κB/AQP8 pathway [5].

In the setting of AP, the structural and functional integrity of mitochondria is of importance not only for cellular oxidative phosphorylation and energy supply but also for the regulation of necrosis [3, 25–28]. Injury-related stress following the attack of AP could damage various calcium pumps and intracellular calcium storage organelles in pancreatic acinar cells, which subsequently lead to intracellular Ca^2+^ overload. This is an early-phase event since the onset of AP and contributes extensively to the progression of the disease. Previously, Xiao et al. reported that AP-related injury and energy stress could be ameliorated by alleviating intracellular Ca^2+^ overload in pancreatic acinar cells [14]. Intracellular Ca^2+^ overload triggers the constant Ca^2+^ influx of mitochondria, which accelerates the loss of MTP due to mitochondrial membrane permeabilization as the result of the opening of the mitochondrial membrane permeability transition pore [29, 30]. These alterations directly lead to irreversible structural and functional damage in mitochondria and finally result in a loss of ATP production and increased necrosis [28, 31–33]. CAMKII acts as a multifunctional Ca^2+^/calmodulin-dependent serine/threonine protein kinase that phosphorylates substrates important in transcription and ion channel regulation in response to increased intracellular Ca^2+^, plays a crucial role in the regulation of the opening of the mitochondrial membrane permeability transition pore although its downstream target is still unknown [34, 35]. The entry of Ca^2+^ into the cell leads to the formation of the Ca^2+^/calmodulin complex in a cooperative form. The Ca^2+^/calmodulin complex binds to the regulatory region of CAMKII and produces a conformational change, which not only phosphorylates its substrates but also leads to autophosphorylation itself to prevent the enzyme from reverting to its inactive conformation and decrease the dissociation rate of the bound calmodulin. Autophosphorylated CAMKII can remain active even when the level of intracellular Ca^2+^ is decreased and therefore acquire autonomous and Ca^2+^ independent activity [16]. Sustained, excessive CAMKII activation is an upstream signaling event for constant intracellular Ca^2+^ overload and mitochondrial Ca^2+^ uptake, inducing the loss of intracellular Ca^2+^ homeostasis [36]. In the present study, our results (Fig. 1) suggested that AP-related necrotic injury was positively regulated by the incubation level of CAMKII. Therefore, CAMKII might be a promising therapeutic target in the future management of AP by maintaining the structural and functional integrity of mitochondria in response to increased levels of intracellular Ca^2+^. MiRs are small noncoding RNAs that bind to mRNAs of target genes at the 3′-UTR, leading to degradation or inhibition of the target mRNAs. MiR, acts as an important mediator in response to various stresses and is involved in signal transductions in response to hypoxia, inflammation and so forth [37–39]. In the present study, an online bioinformatic analysis and a microarray analysis of miRNA using five pairs of pancreatic tissues of rats with or without AP modeling were performed to screen the potential upstream miR that targeted CAMKII. A luciferase reporter assay was then performed and confirmed that miR-30b-5p acts as a negative regulator of CAMKII in our AP models (Fig. 4A, B).

Autophagy acts as a self-aid process by recycling cytoplasmic materials and preserving energy via lysosome-driven degradation in response to various stresses. Regardless of the different potential mechanisms due to the variety of AP models, the impaired autophagy is consistently considered to play a significant role in the pathogenesis of AP [7–10]. Previously, our report suggested that autophagy was overactivated via AMPK/mTOR signaling and positively correlated with AP-related injury in our AP model induced by Na-TC [7]. Nevertheless, several cell death pathways might be simultaneously or sequentially activated since the onset of AP. To our knowledge, the interactions and switches among these cell death pathways are sophisticated in the pathogenesis of AP [13, 25, 40]. Therefore, it might not be rational to explore the mechanisms of AP with one single cell death pathway alone. As stretching research following our previous publications, the present research was performed to determine whether and how impaired autophagy could modulate the expression of CAMKII and regulated necrosis, aiming to elucidate the mechanism of AP more comprehensively through a specific focus on impaired autophagy-regulated necrosis interactions and to shed a new light on the future management of AP. The core autophagic machinery is composed of ATG protein constituents. One particular member of the ATG protein family, ATG7, acts as an E1-like activating enzyme facilitating both LC3-phosphatidylethanolamine and ATG12 conjugation. Thus, ATG7 stands at the hub of these two ubiquitin-like systems involving LC3 and ATG12 in autophagic vesicle expansion to facilitate the final step of autophagosome formation [41]. Given that the impaired autophagy in our AP model was derived from an overactivation of autophagy or so-called increased activity of the upstream autophagic pathway, genetic regulation of ATG7 was introduced into the present study to determine whether and how impaired autophagy could regulate CAMKII-regulated necrosis. First, we confirmed that the activity of overactivated upstream autophagic pathway in our AP model was positively correlated with the incubation level of ATG7 (Fig. 2). Our results indicated that the levels of CAMKII, MTP loss and necrosis of acinar cells after AP induction were all positively correlated with the incubation level of ATG7 (Fig. 3). Given that miR-30b-5p acts as a negative regulator of CAMKII in our AP models, the levels of miR-30b-5p in cells subjected to control, AP, AP + Lv-ATG7 and AP + Lv-sh-ATG7 were measured. The results indicated that ATG7 could negatively regulate the level of miR-30b-5p (Fig. 4C). Hinted by these findings, we hypothesized that there might be a novel pathway, ATG7/miR-30b-5p/CAMKII, that establishes a bridge between autophagy and necrosis and is sophisticated in the pathogenesis of AP. A rescue experiment was therefore designed, and its results suggested that the miR-30b-5p mimic compromised ATG7 overexpression-induced upregulation of CAMKII-regulated necrosis in our AP models (Fig. 5).

As an emerging programmed cell death pathway, regulated necrosis is being studied regarding its pathophysiological involvement and potential clinical relevance in many diseases [3, 4]. Although the mechanisms of AP are still far from known, our results might serve as a preliminary endeavor to unseal Pandora’s box with a specific horizon on impaired autophagy-regulated necrosis interactions. Both miR-30b-5p and CAMKII could potentially be developed as biomarkers that indicate the severity of AP and, furthermore, as readouts that indicate response to the targeted therapy. However, there was a drawback in the present study. The impaired autophagy in our AP model of rats induced by Na-TC was due to the upstream overactivated formation of autophagosomes [7]. Therefore, it is certain that genetic overexpression of ATG7 after AP establishment would lead to more severe impaired autophagy and illness of the disease. However, it is difficult to point out to what extent ATG7 should be silenced when we aim to provide some protection against AP-related damage. ATG7 plays a significant role in the initiation of autophagosome formation. We should not convert the former impaired autophagy derived from the overactivation of upstream formative pathway in response to AP modeling to the latter one derived from insufficient activity of autophagy, given that the basal physiological activity of autophagy in response to AP modeling is indispensable to provide some protection [42, 43]. The ideal range of activated autophagy in response to AP modeling, which may be suggested by some well-quantified parameters, is worthy of elucidation in future studies.

**Fig. 6:**
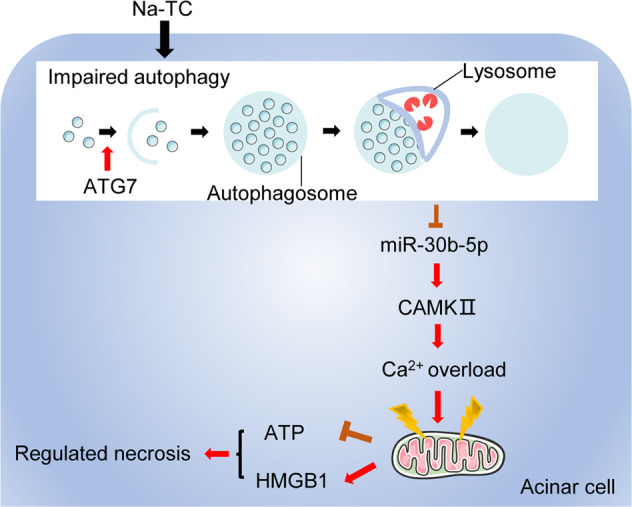
Schematic presentation of the mechanism involved in the promotion of regulated necrosis in response to ATG7-enhanced impaired autophagy in our AP model. AP acute pancreatitis, ATP adenosine triphosphate, CAMKII calcium/calmodulin-dependent protein kinase II, HMGB1 high mobility group protein B1, Na-TC sodium taurocholate.

In conclusion, our results indicated that ATG7-enhanced impaired autophagy exacerbates AP by promoting regulated necrosis via the miR-30b-5p/CAMKII pathway. Some specific studies regarding the uncanonical ATG7/miR-30b-5p/CAMKII pathway might be promising in the future management of AP (Fig. 6).

## Supplementary information

Supplementary Figures Legend

Supplementary Figures

Ethics Approval

aj-checklist

## Data Availability

The datasets generated during the current study are available from the corresponding author on reasonable request.
