# Supplementary material for: ATG7-enhanced impaired autophagy exacerbates acute pancreatitis by promoting regulated necrosis via the miR-30b-5p/CAMKII pathway
Source: Cell Death Dis. 2022 Mar 7;13(3):211. doi: 10.1038/s41419-022-04657-4 (PMC8901675; doi:10.1038/s41419-022-04657-4)
Supplement: Supplementary file 1 — Supplementary Figures Legend [file 41419_2022_4657_MOESM1_ESM.docx]

**Supplementary Figure 1.** Full-length uncropped original Western blots in Fig. 1. The Western blots were double spaced with a wide margin to rule out the possibility that there might be other lanes existed. The Western blots used in the Fig. 1 were marked by a black frame.

**Supplementary Figure 2.** Full-length uncropped original Western blots in Fig. 2. The Western blots were double spaced with a wide margin to rule out the possibility that there might be other lanes existed. The Western blots used in the Fig. 2 were marked by a black frame.

**Supplementary Figure 3.** Full-length uncropped original Western blots in Fig. 3. The Western blots were double spaced with a wide margin to rule out the possibility that there might be other lanes existed. The Western blots used in the Fig. 3 were marked by a black frame.

**Supplementary Figure 4.** Full-length uncropped original Western blots in Fig. 5. The Western blots were double spaced with a wide margin to rule out the possibility that there might be other lanes existed. The Western blots used in the Fig. 5 were marked by a black frame.
