# Supplementary material for: ATG7-enhanced impaired autophagy exacerbates acute pancreatitis by promoting regulated necrosis via the miR-30b-5p/CAMKII pathway
Source: Cell Death Dis. 2022 Mar 7;13(3):211. doi: 10.1038/s41419-022-04657-4 (PMC8901675; doi:10.1038/s41419-022-04657-4)

Supplementary Figure 1

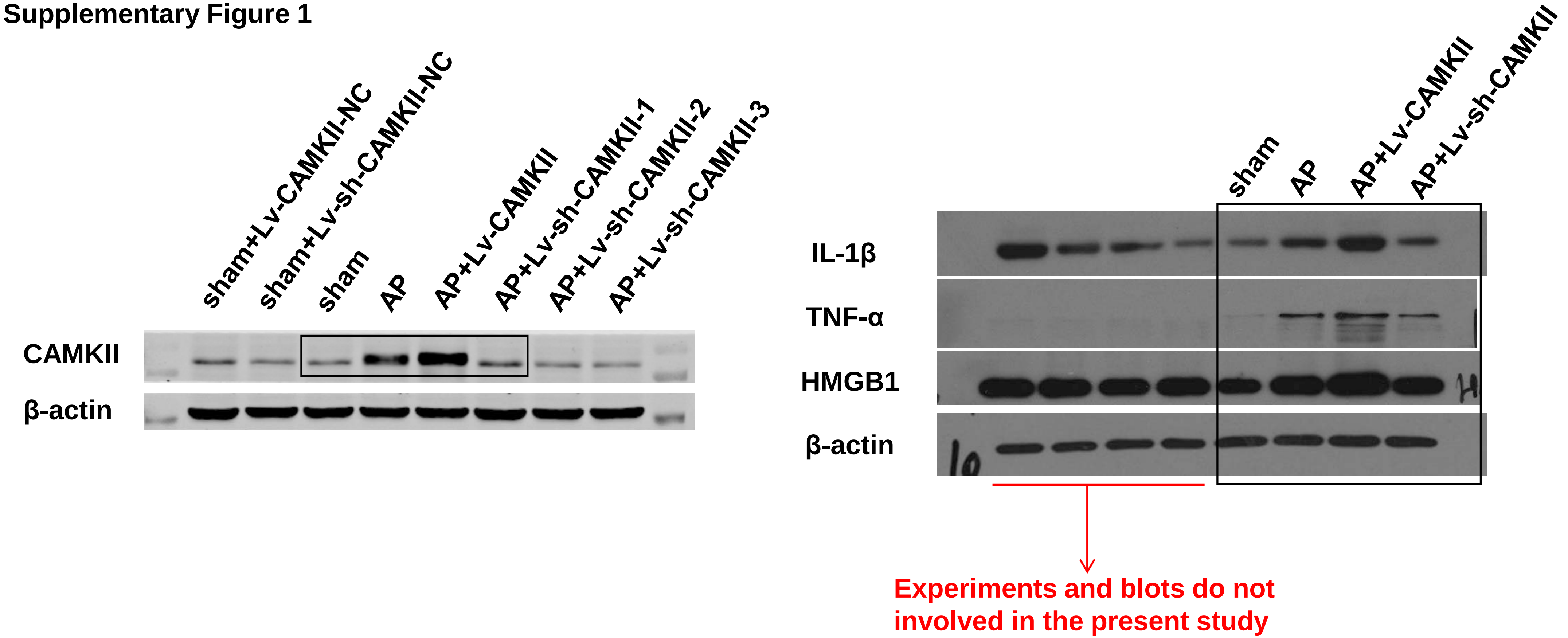

Supplementary Figure 2

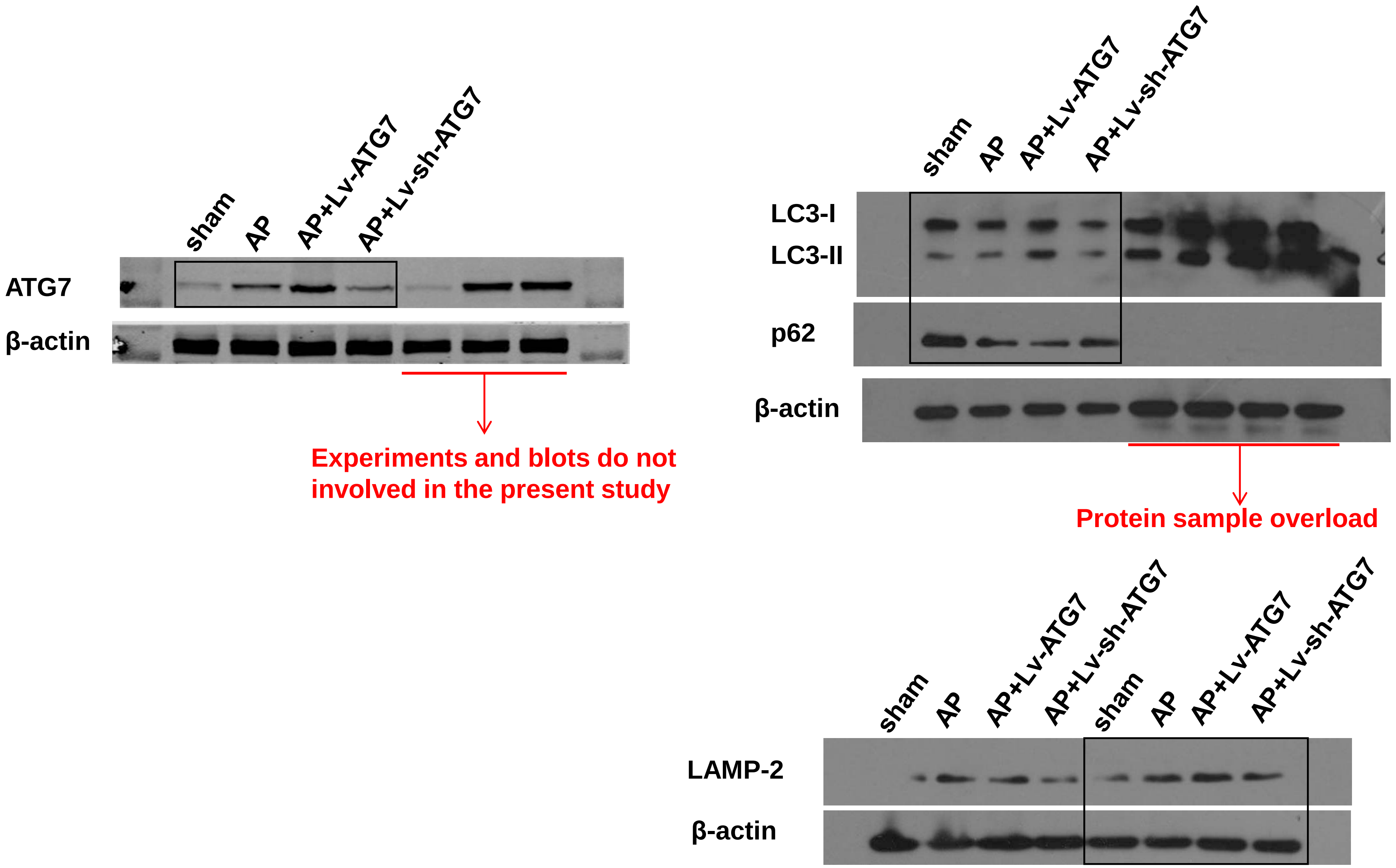

Supplementary Figure 3

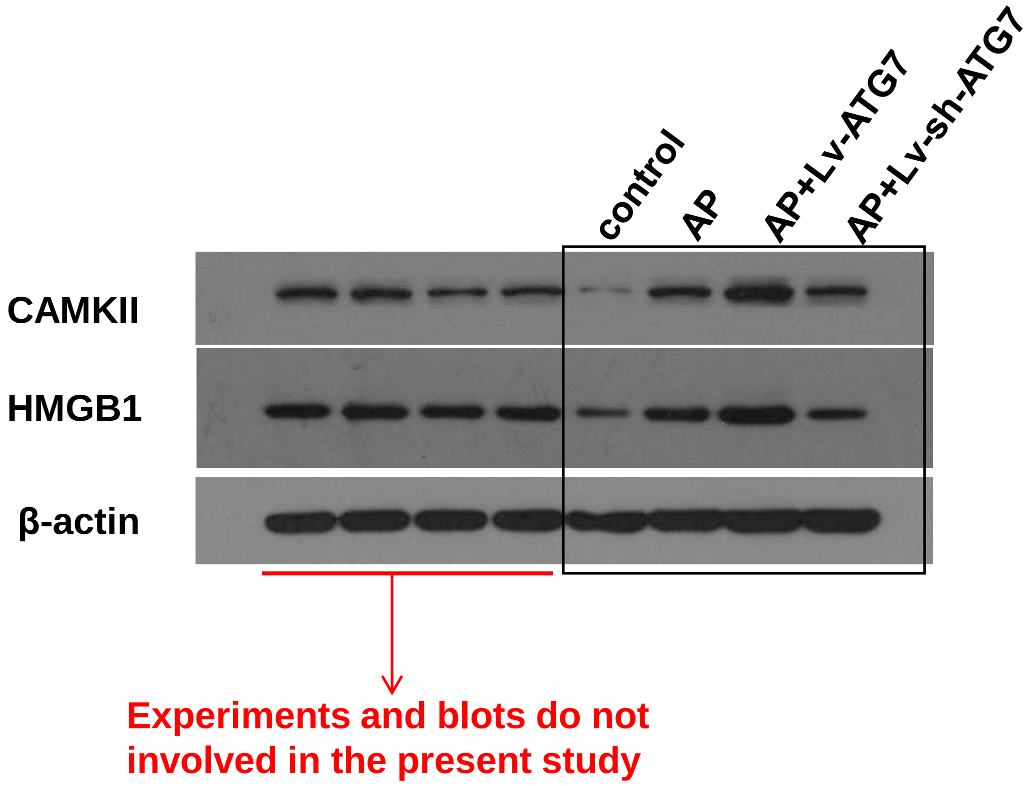

Supplementary Figure 4

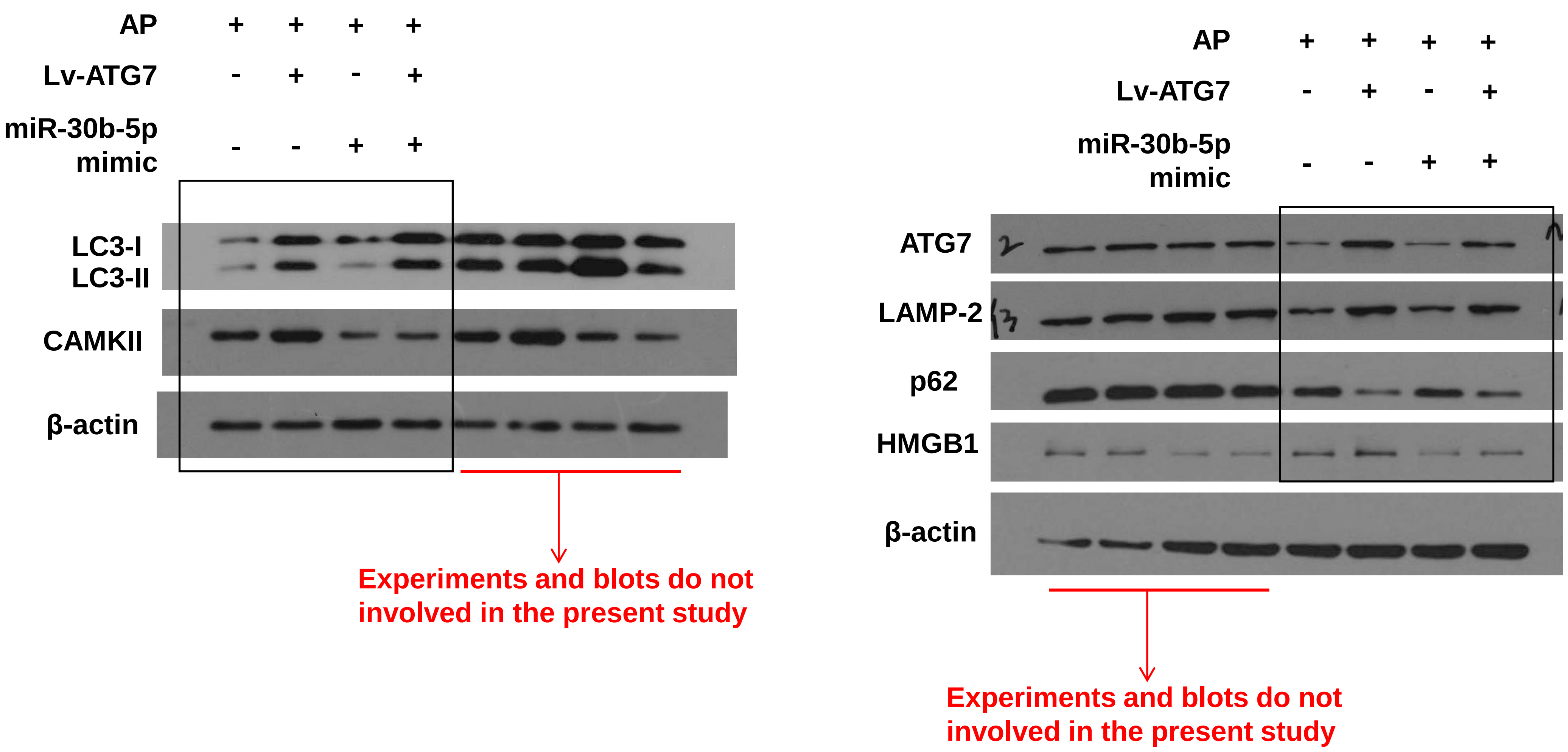

Supplement: Supplementary file 2 — Supplementary Figures [file 41419_2022_4657_MOESM2_ESM.pdf]
