# Supplementary figures and images for: ATG7-enhanced impaired autophagy exacerbates acute pancreatitis by promoting regulated necrosis via the miR-30b-5p/CAMKII pathway
Source: Cell Death Dis. 2022 Mar 7;13(3):211. doi: 10.1038/s41419-022-04657-4 (PMC8901675; doi:10.1038/s41419-022-04657-4)

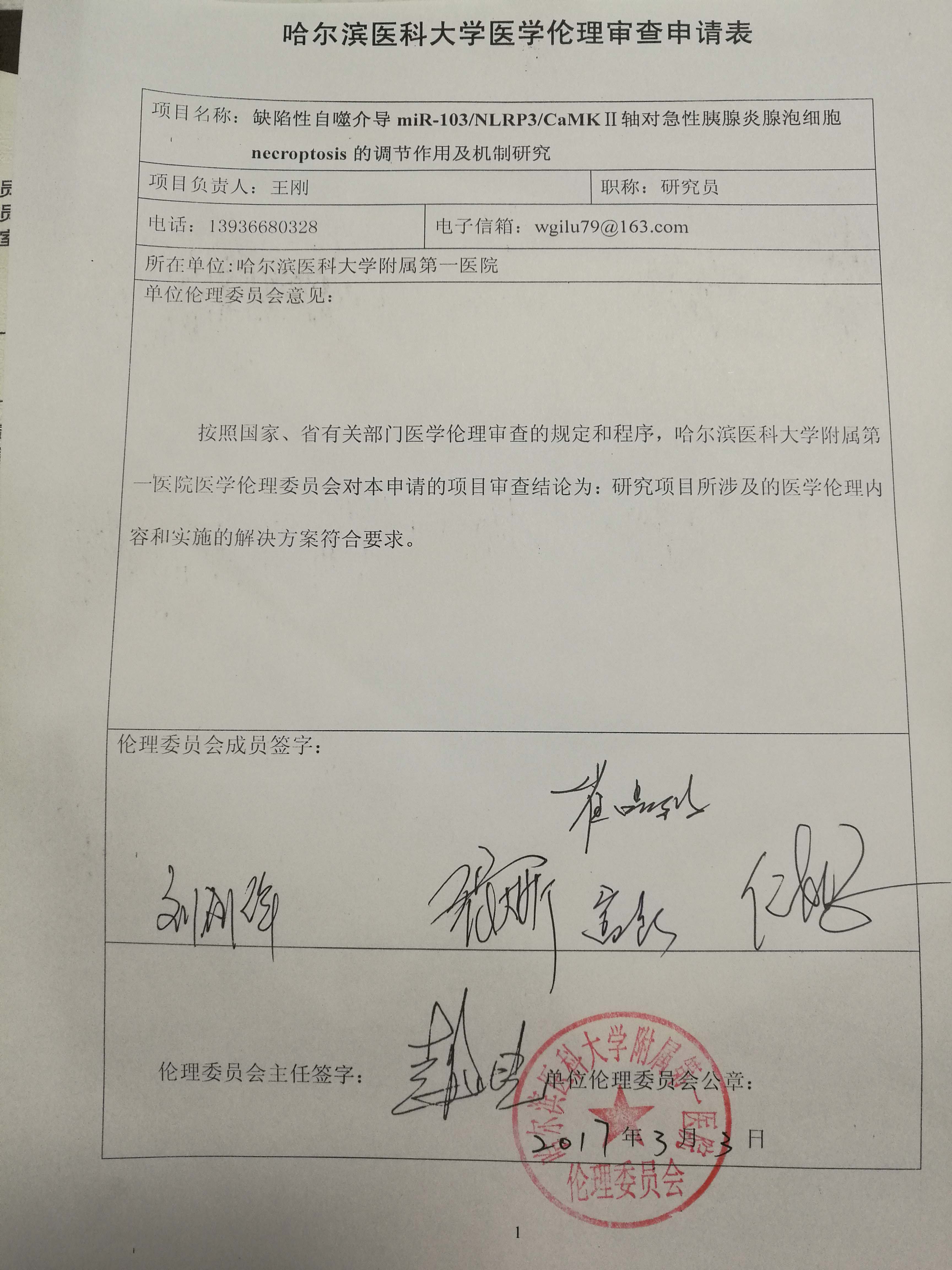

Supplement: Supplementary file 3 — Ethics Approval [file 41419_2022_4657_MOESM3_ESM.jpg]
